# Dietary Intake of Animal and Plant‐Based Protein on Adiposity Measurements and Body Composition in Pre‐ and Postmenopausal Women: A Systematic Review of Randomized Clinical Trials

**DOI:** 10.1096/fj.202600792R

**Published:** 2026-08-01

**Authors:** Sthéfany Nonato Fialho, Maria Luiza de Souza Ferreira, Sarah Aparecida Vieira Ribeiro, Ana Claudia Pelissari Kravchychyn, Helen Hermana Miranda Hermsdorff

**Affiliations:** ^1^ Department of Nutrition and Health, Postgraduate Program in Nutritional Science Federal University of Viçosa Viçosa Minas Gerais Brazil; ^2^ Laboratory of Clinical Analysis and Genomics, Department of Nutrition and Health Universidade Federal de Viçosa (UFV) Viçosa Minas Gerais Brazil; ^3^ Laboratory of Energy Metabolism and Body Composition, Department of Nutrition and Health Universidade Federal de Viçosa (UFV) Viçosa Minas Gerais Brazil

**Keywords:** body mass index, body weight, climacteric, dietary proteins, fat body, weight loss

## Abstract

This systematic review investigated the effect of protein intake on anthropometric and body composition markers in pre‐ to postmenopausal women. Understanding these effects may contribute to the development of nutritional strategies to improve body composition and prevent changes associated with this phase. Searches were conducted in the PubMed, EMBASE, and Cochrane Library databases, following the PRISMA guidelines, and the risk of bias was assessed using the Joanna Briggs Institute (JBI) tool (PROSPERO: CRD420251156313). Fifteen randomized clinical trials, involving 1161 pre‐ and postmenopausal women, were included. Interventions with animal‐based protein (*n* = 8) showed more consistent effects on weight and body fat reduction, especially when combined with calorie restriction and supervised exercise. Protocols using whey protein, meat, or milk protein demonstrated reductions in body fat over 8–16 weeks, with increases in lean mass in longer interventions. However, these effects are strongly influenced by the use of other strategies, such as energy restriction and physical exercise. Studies with soy protein isolate (*n* = 7) showed more heterogeneous results, with few changes in short‐term interventions and more favorable results when combined with exercise. When analyzed independently of energy restriction and physical exercise, the effects of consuming both proteins on body composition were less consistent, making it impossible to confirm benefits from an isolated increase in protein intake. Animal proteins have more consistent effects on anthropometric markers and body composition in women during the climacteric period, particularly when combined with energy restriction and physical exercise. In contrast, plant‐based proteins have more heterogeneous effects, with greater benefits observed when associated with exercise. The findings of this review reinforce the importance of considering protein type, dose, duration of intervention, and other combined strategies when developing nutritional recommendations for changes in body composition.

## Graphical abstract

Graphical summary of the effects of additional intake of animal and plant proteins on anthropometric and body composition parameters in pre‐ and postmenopausal women. The arrows indicate an increase, decrease, or no significant effect.

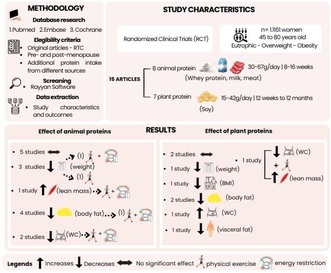

## 1 Introduction

The climacteric period is defined as the period between a woman's reproductive and non‐reproductive phases of life. This period encompasses three phases: perimenopause, characterized by hormonal changes and menstrual irregularity; menopause, determined by the absence of menstrual cycles for at least 12 consecutive months; and postmenopause, which begins after this period and continues until the end of life [1, 2, 3].

**FIGURE 1:**
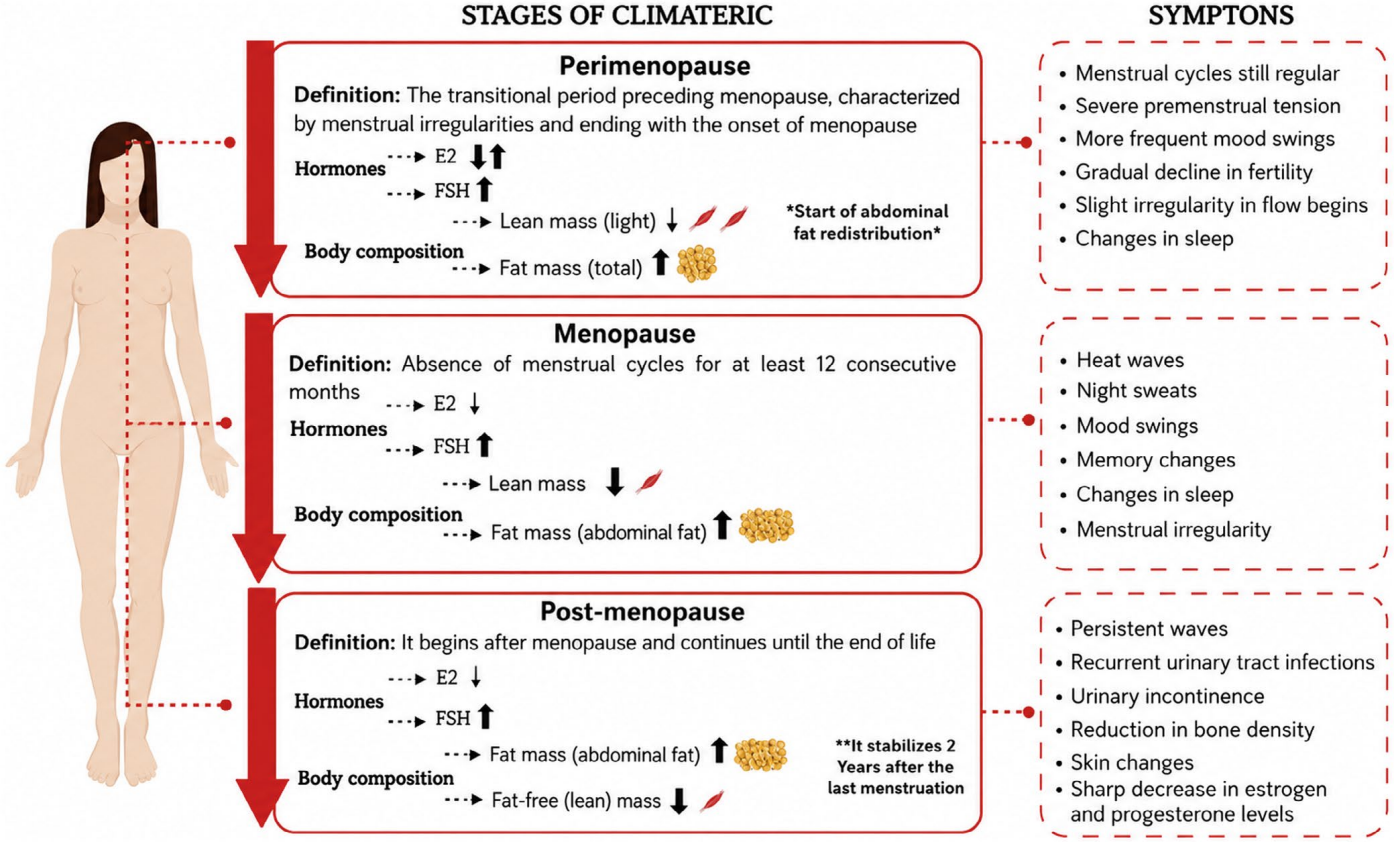
Climacteric stages and physiological‐related changes. Smaller or larger/thicker directional arrows (↑ or ↓) indicate minor or major changes that occur. E2, estradiol; FSH, follicle‐stimulating hormone.

During this period from pre‐ to post‐menopause, there are fluctuations and a sustained reduction in circulating estrogen and neurosteroid levels [4, 5]. These hormonal changes have repercussions on various aspects of women's health, and among the most relevant clinical manifestations in the metabolic sphere is an increase in body fat, especially visceral fat, associated with an increase in abdominal circumference and weight [5]. Figure 1 shows the changes related to each period.

Weight gain, especially increased abdominal adiposity, is strongly associated with the characteristic symptoms of this phase, including hot flashes, night sweats, insomnia, increased fatigue, and reduced quality of life [6]. Thus, the accumulation of visceral fat stimulates the production of pro‐inflammatory cytokines and adipokines, contributing to the onset of a chronic low‐grade inflammatory state in these women [7]. This subclinical inflammatory condition acts as a precursor to hormonal and metabolic changes that affect the neuroendocrine regulation of energy balance, with dysregulation of satiety signaling at gastrointestinal and neurological levels [8, 9].

In this context, adequate food intake and dietary protein quality play a central role in maintaining muscle mass and regulating energy metabolism [10]. However, assessments show that adults consume about 91 g/day of protein, while older individuals consume lower amounts, averaging around 66 g/day [11]. When protein intake is expressed relative to body weight, international guidelines recommend that women in this life stage consume from 1.0 to 1.2 g/kg/day to maintain muscle mass; this intake may reach values from 1.2 to 1.6 g/kg/day for other specific conditions, such as sarcopenia [12, 13, 14, 15]. Therefore, older women often have reduced and inadequate protein intake [10]. Animal proteins provide all amino acids and are well digested [16]. Studies indicate that protein consumption from sources such as dairy products and whey protein (WPP) is associated with reduced muscle mass loss [17, 18]. On the other hand, a plant‐based diet has been associated with a lower risk of developing chronic diseases such as obesity, cardiovascular disease, and diabetes mellitus [19]. Thus, this dietary pattern is motivated by health benefits, concerns related to excessive consumption of animal proteins, especially due to their saturated fat content, as well as environmental, ethical, and sustainability issues [20]. In this sense, plant proteins stand out for providing nutrients such as fiber and polyphenols, and for being produced at lower cost and using fewer resources [21, 22, 23, 24]. However, the quality and source of plant protein can be a limiting factor for muscle mass gain and maintenance, since many have an incomplete essential amino acid profile and great variability in their composition [25].

Despite growing evidence, a comprehensive synthesis remains lacking, as no systematic review has examined the effects of plant and animal protein intake on anthropometric and body composition markers in menopausal women, a period characterized by significant physiological changes. Therefore, this systematic review aims to investigate the effects of animal and plant‐based protein intake on these outcomes in women across the climacteric period, ranging from the pre‐ to the postmenopausal stages.

## 2 Materials and Methods

### 2.1 Protocol and Registration

The review protocol was registered in the International Prospective Register of Ongoing Systematic Reviews (PROSPERO: CRD420251156313), available from https://www.crd.york.ac.uk/PROSPERO/view/CRD420251156313. The review followed the PRISMA (Preferred Reporting Items for Systematic Reviews and Meta‐Analyses) [26].

### 2.2 Search Strategy

Three electronic databases were used for the bibliographic search: PubMed, Embase, and Cochrane Library. The search was conducted in duplicate in October 2024, without applying filters or language restrictions. Controlled descriptors were selected based on Medical Subject Headings (MeSH), Descriptors in Health Sciences (DeCS), and Emtree terms in English. Boolean operators OR and AND were used to connect terms during the search. The search strategy included the terms (Menopause OR Premenopause OR Perimenopause OR Postmenopause OR Climacteric) AND (Plant Proteins, Dietary OR Animal Proteins, Dietary) AND (Body Weight OR Waist Circumference OR Waist‐Hip Ratio OR Body Fat Distribution OR Adipose). Tissue, along with their synonyms, abbreviations, and plurals, according to each database (Table S1).

### 2.3 Eligibility Criteria

Clinical trials with women in climacteric periods that evaluated the effect of consuming animal and plant protein sources on anthropometric markers and body composition were eligible for review. To guide the selection of studies and define the main research question, we followed the PICOS approach (*P* = population; I = intervention; C = comparison; O = outcomes; S = study design). Therefore, the guiding question of our review was: “In pre‐ and postmenopausal women, does the consumption of different sources of animal or plant protein promote benefits in anthropometric markers and body composition?”. Table 1 outlines the specific criteria applied.

**TABLE 1:** PICO‐based inclusion and exclusion criteria for study selection.

Parameter	Inclusion criteria	Exclusion criteria
Population	Women in pre‐, peri‐, and post‐menopause (climacteric) who have health weight, overweight, or obesity	Teenagers, children, men, or any other age group and gender
Intervention	Additional consumption of dietary or supplemental proteins from different sources	Any other type of study that does not evaluate protein intake
Comparison	Placebo, normal protein diet, low protein diet, or control group without supplementation or additional protein consumption.	Studies analyzing to carbohydrate and fat consumption, not related to protein consumption
Outcomes	Anthropometric markers (Weight loss, waist circumference, hip circumference, waist‐to‐hip ratio—WHR), and body composition (body fat, visceral fat, and lean mas)	Studies that do not evaluate anthropometric and body composition results or only evaluate biochemical/metabolic outcomes
Study design	Randomized clinical trials	Observational studies (cohort, case–control, cross‐sectional), narrative or systematic reviews, meta‐analyses, animal studies, case reports, editorials and letters to the editor, guidelines, and consensus statements

### 2.4 Screening and Selection of Articles

The studies found in the databases were exported to Rayyan QCRI software. The selection of articles was performed independently and blindly by two reviewers (S.N.F. and M.S.F.). In the first stage, duplicates were removed, followed by screening titles and abstracts to identify studies eligible for full reading. Disagreements after reading the full text were resolved with the participation of a third reviewer (A.C.P.K.). Full‐text was accessed by CAPES institutional resources (https://www‐periodicos‐capes‐gov‐br.ez35.periodicos.capes.gov.br).

### 2.5 Data Extraction

After final selection of the studies to be included, a standardized data‐extraction table was developed, defined by mutual agreement among the authors. The extraction was performed independently by two researchers (S.F.N. and M.S.F.). The extracted variables of interest included: author, year of publication, study design, sample characteristics (*n*, age, climacteric period, and nutritional status), intervention (protein type), physical activity practice, and method of body composition analysis. The second table extracted: intervention, form of protein intake, intervention monitoring, use of hormone replacement therapy, and outcomes. For the nutritional status variable presented in the results extraction table, the inclusion criteria reported by each study were initially adopted. When this information was not explicitly stated, nutritional status was defined based on the body mass index (BMI) values described in the sample characterization. The following reference ranges were used for this classification: BMI 18.5–24.9 kg/m^2^ (health weight), 25.0–29.9 kg/m^2^ (overweight), and ≥ 30 kg/m^2^ (obesity). In addition, when the protein content of the foods was not reported, it was estimated using the Brazilian Food Composition Table (TACO) (Universidade Estadual de Campinas, 2011), based on the nutritional values corresponding to the portions used in the intervention [27]. Two authors (S.F.N and M.S.F) extracted the data and reviewed the information to ensure accuracy.

### 2.6 Assessment of Risk of Bias

The studies that were included by both authors (S.N.F. and M.S.F.) were assessed individually, independently, and in parallel. Any discrepancies were resolved by mutual agreement. The analysis was performed using the Joanna Briggs Institute (JBI) tool [28, 29]. Studies were classified as low (> 70%), moderate (50%–70%), or high risk of bias (< 50%) based on the percentage of affirmative responses [30].

## 3 Results

### 3.1 Characteristics and Selection of the Studies

**FIGURE 2:**
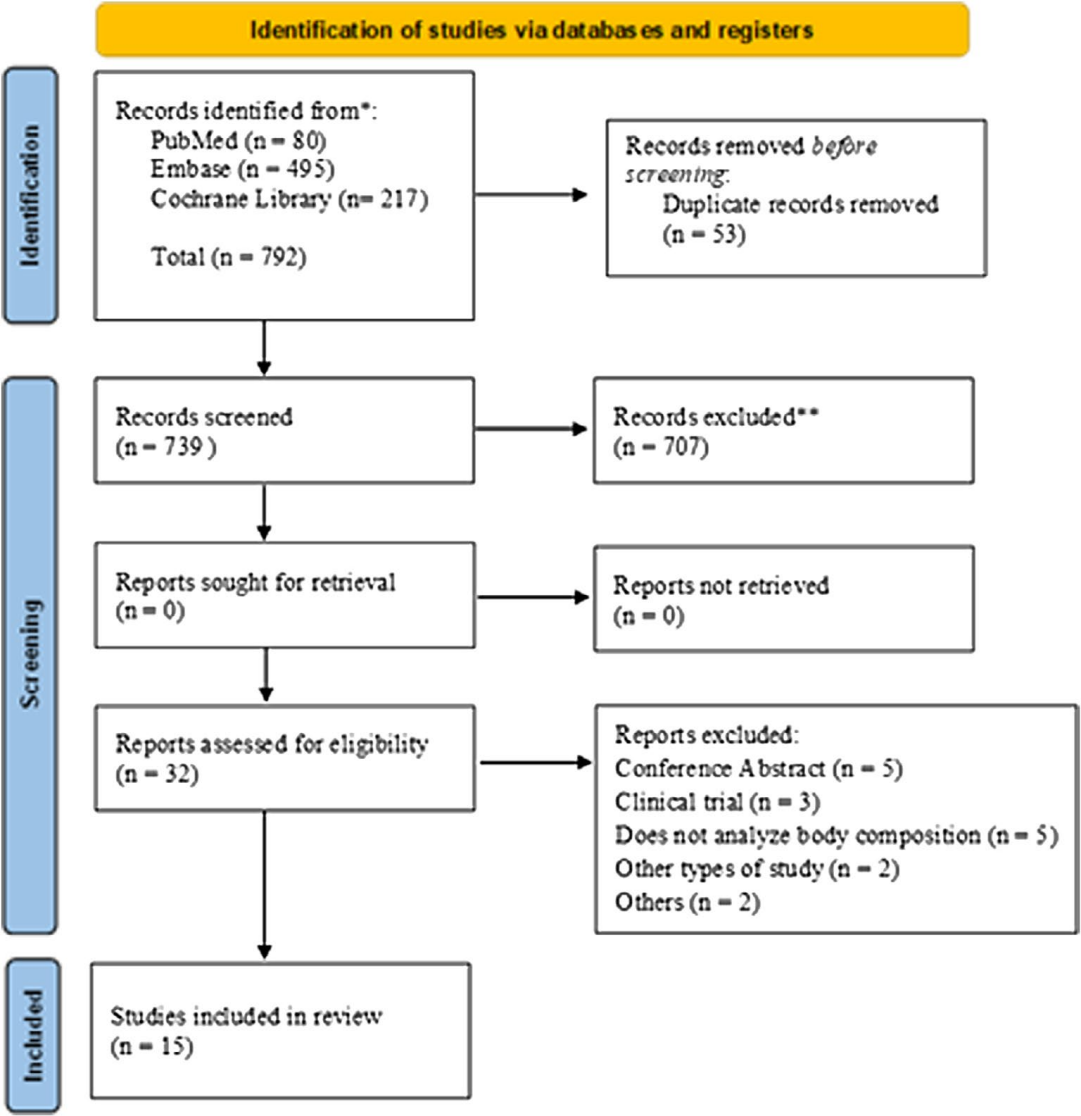
PRISMA flowchart of the literature search process and study selection.

A total of 792 references were retrieved from the databases. After removing duplicates, 739 references remained. During screening of the title and abstract, 707 records were excluded based on the initial exclusion criteria. Of the 32 titles accepted for full‐text reading, 17 were excluded because they did not meet the inclusion criteria. Finally, 15 studies were included in this systematic review (Figure 2).

This systematic review included 15 studies conducted between 2001 and 2020 (Table 2), all of which were randomized controlled trials (RCTs). Seven of the articles were conducted in the United States, two in Canada, one in California, one in Brazil, one in China, one in the Netherlands, one in Australia, and one in Iran.

**TABLE 2:** Methodological characteristics of the included studies (*n* = 15).

Reference, country	Study type	Sample characteristics	Intervention	Physical activity	Anthropometric and body composition analysis
Garden et al. [31]; USA	RCT Double‐blind Placebo‐controlled	*n* = 94 Age: < 80 years Postmenopausal Nutritional status: Healthy weight, Overweight and Obesity	Soy vs. Milk protein	Not specified	Weight
Moeller et al. [32]; USA	RCT Double‐blind Placebo‐controlled	*n* = 69 Age: 50.6 years Perimenopausal Nutritional status: Healthy weight and Obesity	Soy vs. Whey protein	Those who engaged in excessive physical exercise were excluded [expending > 10 460 kJ (2500 kcal) per week]	DXA
Kok et al. [33]; USA	RCT Double‐blind Placebo‐controlled	*n* = 202 Age: 60–75 years Postmenopausal Nutritional status: Overweight	Soy vs. Milk protein	Not specified	BMI, waist‐to‐hip ratio
McFarlin et al. [34]; USA	RCT	*n* = 54 Age: 50–80 years Postmenopausal Nutritional status: Overweight and Obesity	Beef vs. Chicken vs. Carbohydrate vs. Usual diet	Usual activity	Air‐displacement plethysmography
Maesta et al. [35]; Brazil	RCT Double‐blind Placebo‐controlled	*n* = 46 Age: 45–70 years Postmenopausal Nutritional status: Overweight	Soy vs. Maltodextrin	Progressive resistance training, 3 times/week for 16 weeks, including 8 exercises (3 sets of 8–12 repetitions)	BIA WC.
Mahon et al. [36]; USA	RCT Double‐blind Placebo‐controlled	*n* = 54 Age: 58 Postmenopausal Nutritional status: Overweight	Carbohydrates vs. Beef vs. Chicken vs. Usual diet	Usual activity	DXA
Sites et al. [37]; USA	RCT Double‐blind Placebo‐controlled	*n* = 15 Age: 55.6 years Postmenopausal Nutritional status: Obesity	Soy vs. Casein	Regular physical exercise (> 2 times/week) was excluded.	DXA
Liu et al. [38]; China	RCT Double‐blind Placebo‐controlled	*n* = 166 Age: 48–70 years Postmenopausal Nutritional status: Overweight and obesity	Soy vs. Whey	Usual activity	BIA
Mojtahedi et al. [39]; USA	RCT Parallel design Double‐blind Placebo‐controlled	*n* = 31 Age: 65.2 years Postmenopausal Nutritional status: Overweight and Obesity	Whey protein vs. Maltodextrin	2–3×/week, stretching + walking, same protocol for IG and GC	DXA
Josse et al. [40]; Canada	RCT Placebo‐controlled	*n* = 81 Age: 19–45 years Postmenopausal Nutritional status: Overweight and Obesity	IG1 (15% milk protein) vs. IG2 (7.5% milk protein) vs. IG3 (< 2% milk protein) + Calorie deficit: −500 kcal	Supervised exercise 7×/week (5 supervised, 2 unsupervised): daily aerobic (~250 kcal/session) + resistance training 2×/week	DXA
Van nielen et al. [41]; Netherlands	RCT Crossover Controlled diet clinical trial	*n* = 15 Age: 45–70 years Postmenopausal Nutritional status: Abdominal Obesity (stable body weight)	Soy vs. Mixed protein sources	Usual activity	DXA
Weisgarber et al. [42]; Canadá	RCT Double‐blind Placebo‐controlled	*n* = 12 Age: 57 years Postmenopausal Nutritional status: Overweight	Whey protein vs. Placebo	Unilateral resistance training 4 days a week. Each participant trained one side with whey and the other with a placebo.	DXA
Zhu et al. [43]; Australia	RCT Double‐blind Placebo‐controlled	*n* = 196 Age: 70–80 years Postmenopausal Nutritional status: Overweight	Whey protein vs. Milk	Not specified	DXA
Smith et al. [44]; USA	RCT	*n* = 70 Age: 50–65 years Postmenopausal Nutritional status: Obesity	0.8 g/kg/day maintenance vs. 0.8 g/kg/day hypocaloric diet vs. 1.2 g/kg/day whey protein	Could not be involved in physical activities ≥ 1.5 h of exercise per week	DXA and MRI
Haidari et al. [45]; Iran	RCT Double‐blind Placebo‐controlled	*n* = 56 Age: 50–65 years Premenopausal Nutritional status: Obesity	Whey Protein (−912 kcal) vs. Normal Diet (−800 kcal)	IPAQ (low, moderate, high).	BIA WC

Abbreviations: BIA, bioimpedance; BMI, body mass index; DXA, dual‐energy X‐ray absorptiometry; IPAQ, International Physical Activity Questionnaire; MRI, magnetic resonance imaging; RCT, randomized clinical trial; WC, waist circumference.

The total sample comprised 1161 women aged 45–80 years. Among the studies, 13 included participants with overweight or obesity, and 2 included women with healthy‐weight, overweight, or obesity. Of the included studies, 13 evaluated postmenopausal women, one evaluated premenopausal women, and one evaluated perimenopausal women.

Regarding protein supplementation, seven studies evaluated plant‐based proteins, specifically soy protein, while eight investigated animal‐based proteins, including whey protein and meat consumption, with interventions ranging from 8 weeks to 2 years. The plant‐based protein (soy) interventions ranged from 15 to 42 g/day. Of the seven studies included with this protein source, one combined the intervention with resistance exercise. As for the control groups, two used milk protein, two used whey protein, one used maltodextrin, one used casein, and one used mixed protein sources without soy. In six studies, protein was administered as a supplement, and in one, as food. Interventions with animal protein also varied widely in dose, ranging from 30 to 67 g/day. Of the eight studies that used this protein source, four are energy‐restricted interventions, and two are combined with physical exercise. With regard to the control groups, three studies used a habitual diet, one used maltodextrin, another used maltodextrin combined with sucralose, and another used a skim milk‐based beverage. Protein intake was provided in the form of a supplement in six studies and through dietary sources in two studies, as summarized in Table 3.

**TABLE 3:** Description of the interventions according to protein type, use of hormone replacement therapy, and body composition outcome.

	Intervention (type of protein/dose)	Form of protein intake and duration	Monitoring the intervention	Outcomes
Garden et al. [31]; USA	IG1: Soy^−^: soy protein with trace amounts of isoflavones (42 g/day) (*n* = 33) IG2: Soy^+^: soy protein with 80 mg of isoflavones (42 g/day) (*n* = 31) CG: Milk protein (42 g/d) (*n* = 30)	Supplementation 16 weeks (4‐week adaptation +12‐week intervention)	Counting the number of empty supplement containers returned at each bimonthly clinical consultation.	↔ Weight
Moeller et al. [32]; USA	IG1: Isoflavone‐rich soy protein isolate (SPI^+^)—40 g/day of isoflavone‐rich soy protein (*n* = 24) IG2: Isolated soy protein low in isoflavones (SPI^−^)—40 g/day of soy protein with low isoflavone content (*n* = 24) CG: Whey protein—40 g (*n* = 21)	Supplementation 24 weeks	Food records and urine samples for urinary isoflavone analysis.	↔ Body mass, body fat, weight, WC, HC between groups. ↑ HC (IG1)
Kok et al. [33]; USA	IG: Solae (36.5 g of the product) 25.6 g/day of soy protein (*n* = 100) CG: Placebo composed of milk protein 25.6 g/day (*n* = 102)	Supplementation 12 months	Food Diary and QFA. Assessment of plasma genistein levels in the blood sample from the last consultation.	↔ BMI, waist‐to‐hip ratio between groups.
McFarlin et al. [34]; USA	Low‐calorie diet (≈1250 kcal/day) for 9 weeks + IG1: Beef: 26% protein (67 g/day), 48% carbohydrate, 26% fat (*n* = 14) IG2: Chicken: 25% protein (67 g/day), 51% carbohydrate, 24% fat (*n* = 15) IG3: Carbohydrate: 16% protein (51 g/day), 59% carbohydrate, 24% fat (*n* = 14) GC: Usual diet, without restriction (*n* = 11)	Supplementation 11 weeks (2‐week adaptation +9‐week intervention)	Food Diary	↓ Weight and body fat (IG1, IG2, IG3) ↔ body weight and body fat between groups ↔ lean mass.
Maesta et al. [35]; Brazil	IG1: Soy protein (25 g/dia) containing 50 mg isoflavones—32 mg genistein, 15 mg daidzein and 3 mg glycitein (*n* = 10) IG2: Soy protein (25 g/dia) containing 50 mg of isoflavones—32 mg genistein, 15 mg daidzein and 3 mg glycitein + resistance exercise (*n* = 14) CG1: 25 g/dia of maltodextrin (*n* = 11) CG2: 25 g/dia of maltodextrin + resistance exercise (*n* = 11)	Supplementation 16 weeks	Urinary isoflavone dosage by HPLC	↔ BMI, body fat (%) in the groups. ↓ waist circumference (IG2 and CG2) ↑ body mass (IG2 and CG2).
Mahon et al. [36]; USA	Basal diet 1000 kcal/day + IG1: Carbohydrates (butter cookies and chocolates covered with sugar—250 kcal/day) (*n* = 14) IG2: Beef (the groups received 250 kcal/day of cooked filet mignon—250 kcal/day ≈33 g of protein) (*n* = 14) IG3: Chicken (chicken breast cooked with butter—250 kcal/day ≈12 g of protein) (*n* = 15) CG: Usual diet (*n* = 11)	Food (11 weeks—weeks of weight maintenance +9 weeks of dietary intervention and energy restriction)	Food was delivered weighed to participants; Three‐day food record; Urinary nitrogen excretion	↓ Weight loss (IG1, IG2, IG3), ↔ between groups. ↓ body fat (IG1, IG2, IG3), ↔ between groups, ↑ body fat (CG) ↓ lean mass (IG1, IG2, IG3), ↔ between groups.
Sites et al. [37]; USA	IG: Daily shake containing soy (20 g of soy protein plus 160 mg of isoflavones) (*n* = 9) CG: Daily isocaloric casein placebo shake (20 g of casein protein) (*n* = 6)	Supplementation 3 months	Not specified	↔ Weight, BMI, fat body, fat mass, visceral fat between IG e CG ↓ abdominal fat (IG).
Liu et al. [38]; China	IG: 15 g of soy protein and 100 mg of isoflavones (*n* = 57) CG1: 15 g of whey protein and 100 mg of isoflavones (*n* = 58) CG2: 15 g of whey protein (*n* = 51)	Supplementation 6 months	Counting sachets at in‐person meetings 3‐day food diary	↑ Weight loss (IG); ↓BMI and body fat (IG).
Mojtahedi et al. [39]; USA	IG: 50 g/day whey protein isolate + calorie deficit 500 kcal (*n* = 15) CG: 50 g/day maltodextrin + calorie deficit 500 kcal (*n* = 16)	Supplementation 6 months	Monthly online food records Empty packaging was collected	↑ Weight loss, subcutaneous and intermuscular fat ↓ lean mass (IG, CG), ↔between the groups.
Josse et al. [40]; Canada	IG1: 15% of energy from milk protein (6–7 servings/day); macronutrient distribution 40% CHO, 30% LIP, 30% PTN, with total protein doubled compared to other groups—56/g/day of protein (*n* = 27) IG2: 7.5% of energy from milk protein (3–4 servings/day), macronutrient distribution 55% CHO, 30% LIP, 15% PTN 29 g/day of protein (*n* = 30); IG3: < 2% of energy from milk protein (0–1 serving/day); macronutrient distribution 55% CHO, 30% LIP, 15% PTN—8 g/dia of protein (*n* = 24) + Calorie deficit: −500 kcal	Food 16 weeks	3‐day food diary	↓ BMI e waist circumference (IG1, IG2, IG3), ↔ between the groups; ↓visceral fat (>IG1 and IG3); ↑ lean mass (IG1), ↔ (IG2), ↓ (IG3); ↓ body fat (IG1, IG2, IG3), > IG1.
Van Nielen et al. [41]; Netherlands	IG: Normocaloric diet with partial replacement of meat protein by ≈30 g/day of soy protein (22% of total energy) (*n* = 15) CG: Isocaloric diet with mixed protein sources (mainly meat, dairy, and bread) without soy (*n* = 15)	Food 8 weeks	Consumption was in the presence of a supervisor, and when that was not possible, the meal was weighed	↓ Total and abdominal fat; ↔ between diets.
Weisgarber et al. [42]; Canada	IG: Whey protein—40 g/day of ISOWhey Breezer (lemon iced tea–flavored powder; Interactive Nutrition, manufactured by NutrMix)—during unilateral training (*n* = 12) CG: Placebo—Corn starch maltodextrin (30 g) + sucrose (10 g), lemon iced tea flavor—during training of the contralateral limb (*n* = 12)	Supplementation 10 weeks	Consumption in the presence of a supervisor.	↔ Lean mass; ↑ RM
Zhu et al. [43]; Australia	IG: 250 mL/day of a high‐protein beverage (30 g whey protein isolate; 600 mg calcium; 3.2 kJ/mL) (*n* = 101) CG: 250 mL/day of a skim milk–based beverage with identical calories and calcium to the intervention (2.1 g of protein) (*n* = 95)	Supplementation 2 years	3‐day food diary (2 weekdays and 1 weekend day)	First Year: ↑ Weight, lean mass (IG and CG); ↓ muscle area of the arm (IG). Second year: ↔ weight, lean mass, muscle area of the arm.
Smith et al. [44]; USA	IG1: weight maintenance—0.8 g/kg/day (*n* = 18) IG2: weight loss—low‐calorie diet—0.8 g/kg/day (*n* = 27) IG3: weight loss with protein supplementation—1.2 g/kg/day—whey‐protein isolate (*n* = 25)	Supplementation 6 months	Daily food record; blood urea nitrogen and, in a subgroup of participants, urinary urea nitrogen excretion were measured as objective markers of protein intake.	↔ Lean mass and body fat.
Haidari et al. [45]; Iran	IG: Isocaloric diet for weight loss (−912 kcal) + whey protein (30 g/day) (*n* = 27) CG: low‐calorie diet (−800 kcal)—55% carbohydrates, 30% lipids, 15% proteins—no supplementation (*n* = 29)	Supplementation 8 weeks	Contact with a nutritionist; 24‐h dietary recall (2 weekdays and 1 weekend day) at the beginning and end of the study.	↓ Weight and BMI (IG and CG); ↓ WC, body fat (IG) ↑ lean mass (IG).

*Note:* ↓ = decrease; ↑ = increase; ↔ = not significant (*p* > 0.05).

Abbreviations: CG, control group; HC, hip circumference; IG, intervention group; RM, repetition maximum.

Body composition assessment involved direct and indirect methods. Nine studies used DXA, three used BIA, and one used a 4‐compartment model; four combined this method with another. Anthropometric outcomes were also reported, including body weight in one study and BMI in one study. Regarding physical activity analysis, seven interventions used specific exercises, eight of which assessed only habitual activity or analyzed activity levels.

### 3.2 Effects of Protein Intake on Body Composition

#### 3.2.1 Effect of Protein Type, Protein Dose, and Intervention Duration

In interventions involving animal‐derived protein, doses range from 30 to 56 g/day from whey protein or milk [39, 40, 42, 43, 45] and between 30 and 67 g/day from meat [34, 36]. Additionally, Smith et al. (2018) [44] compared intakes of 0.8 g/kg/day and 1.2 g/kg/day associated with whey protein supplementation, making it the only study to express protein intake relative to body weight.

Regarding intervention duration, dietary interventions involving animal protein ranged from 8 to 16 weeks, except for [43], who conducted their study over a two‐year period. Over relatively short periods, three studies [36, 40, 41] reported consistent reductions in body fat, suggesting that brief dietary interventions with animal proteins (meat or milk) may improve the body composition of postmenopausal women; however, these results should be interpreted with caution to account for the presence of other important factors, such as energy restriction and exercise protocols.

When analyzing the outcomes specifically, the intake of 40 g/day of whey protein over 16 weeks, combined with resistance training, increased strength but did not change lean body mass [42]. In turn, [43] y protein, followed by stabilization in the second year. Another 8‐week intervention with 30 g of whey protein combined with energy restriction reduced weight, BMI, waist circumference, and body fat, and increased lean body mass in the supplemented group [45]. Finally, Smith et al. [44]. observed no significant differences in body composition changes between protein intakes of 0.8 g/kg/day and 1.2 g/kg/day over a 6‐month period across both weight maintenance and weight loss conditions, suggesting that increasing protein intake alone may not be the primary driver of changes in lean mass and body fat.

In general, interventions involving higher doses or longer durations, such as those reported by Garden et al. (2001) [31], who administered 42 g/day for 16 weeks, and [32], who administered 40 g/day for 24 weeks, showed no changes in weight, body fat, or hip circumference. Similar results were demonstrated by [33], even in a prolonged intervention, where they provided 25.6 g/day for 12 months and also found no changes in BMI or waist‐to‐hip ratio. On the other hand, shorter interventions showed more heterogeneous results, with some studies presenting more specific findings, such as [37], who administered 20 g/day for 3 months and found no changes in BMI but observed a reduction in abdominal fat in the group that consumed soy. In a 6‐month study [38], 15 g/day of soy protein with isoflavones was administered, resulting in a reduction in BMI and body fat. A 16‐week intervention provided 25 g/day [35] and identified a reduction in waist circumference.

It is important to note that differences in the amounts of animal and plant‐based proteins, as well as the lack of standardized intake relative to body weight, may have influenced the observed results. As such, it is difficult to make a direct comparison between protein sources and to identify the dose–response relationship.

Although a meta‐analysis was considered for this systematic review, it was not possible to conduct one due to the significant heterogeneity among the included studies. There was variation in methodological design, study populations, and data collection instruments. Furthermore, not all body composition and anthropometric markers were assessed across all studies, making it impossible to calculate pooled effect estimates. Finally, the results were inconsistent and not replicable.

#### 3.2.2 Effects of Protein Intake With and Without Calorie Restriction

Of the 15 studies included, six used animal protein sources in conjunction with an energy restriction [34, 36, 39, 40, 44, 45]. In contrast, of the seven studies that evaluated plant‐based proteins, none were conducted under energy restriction [31, 32, 33, 35, 36, 37, 38, 41].

Josse et al. [40] reported reductions in body fat over relatively short periods, ranging from 11 to 16 weeks. However, these interventions were conducted under energy restriction, which is an important confounding factor to note. Thus, the observed reductions in body fat cannot be explained solely by animal protein intake, since energy restriction also plays an important role in improving body composition.

Similarly, in studies that employed low‐calorie diets and included animal protein [34, 36], consuming approximately 67 g/day of beef or chicken for 9 weeks resulted in both weight loss and reduced body fat, with no differences between the protein sources consumed, reinforcing the role of energy restriction as a key determinant of fat loss.

[39] also conducted their study under conditions of energy restriction and observed greater weight loss and reductions in subcutaneous and intramuscular fat through whey protein supplementation; however, both groups experienced a reduction in lean body mass. In contrast, [43], in their 2‐year intervention without energy restriction, observed an initial increase in body weight and lean mass, suggesting a distinct response when energy restriction is not applied.

Also noteworthy is the study by [44], which compared animal protein intake at two levels (0.8 g/kg/day and 1.2 g/kg/day) under different conditions—maintenance and weight loss. In this context, the authors observed no differences in lean body mass and body fat between the groups evaluated, regardless of protein intake, indicating that energy restriction has a greater influence on body composition than an isolated increase in protein consumption.

Finally, [45], in a weight‐loss intervention involving whey protein supplementation, reported reductions in weight, BMI, waist circumference, and body fat, as well as an increase in lean body mass. However, since the study also involved calorie restriction, it is not possible to determine the effect of protein intake on these outcomes.

#### 3.2.3 Effects of Protein Consumption Combined With and Without Physical Exercise

Of the 15 studies included, three combined the intervention with physical exercise; two of these were animal‐based [40, 42] and one was plant‐based [35]. The inclusion of exercise alongside the protein intervention varied across the analyzed studies, standing out as a significant confounding factor regarding body composition.

[42] stand out for combining supplementation with 40 g/day of whey protein with a supervised unilateral resistance training protocol over 10 weeks, observing an increase in strength but no changes in lean body mass among participants, which suggests that exercise influences these outcomes independently of changes in body composition. Similarly, [40] evaluated supplementation with 56 g/day of milk protein and used a supervised exercise protocol with a frequency of 7 times per week (5 supervised and 2 unsupervised sessions), which should be considered a relevant factor in interpreting the results, making it difficult to attribute the effects solely to protein intake.

Furthermore, [35] examined the intake of 25 g/day of soy protein in combination with resistance training over a 16‐week period, which resulted in a reduction in waist circumference. However, the inclusion of training makes it impossible to determine the isolated effect of protein intake on body composition.

On the other hand, several studies on soy protein [31, 32, 33, 41, 43] did not include structured exercise protocols, evaluating only the dietary intervention, which truly reflects the effect of protein intake. In general, in these studies, no changes were observed in weight, lean body mass, body fat, or BMI.

Among studies that did not include exercise intervention, [38], in a 6‐month trial involving soy protein supplementation combined with isoflavones, observed reductions in weight, BMI, and body fat. In another shorter‐term intervention [37], also involving the use of isoflavones, a reduction in abdominal fat was observed after 3 months, with no changes in the other parameters.

Overall, the findings suggest that the presence of exercise protocols represents a significant confounding factor in interpreting the effects of protein intake on body composition, making it difficult to distinguish between the isolated effects of diet and those resulting from training. In studies that combined protein supplementation with resistance training, the positive outcomes observed, such as increased strength and reduced anthropometric measurements, may be more closely related to the combination of the mechanical stimulus of exercise with protein intake rather than to protein intake alone.

### 3.3 The Stages of Pre‐ and Post‐Menopause and the Use of Hormone Replacement Therapy

Of the included studies, 13 were conducted with postmenopausal women, the group with the most research on protein intervention. In contrast, only one study was conducted in premenopausal women [45] and one in perimenopausal women [32]. Thus, the distribution of studies indicated a greater concentration of evidence in the postmenopausal phase, with less representation of the premenopausal and perimenopausal phases. Regarding hormone replacement therapy, 14 studies reported that participants were not undergoing hormonal therapy, while one study did not specify whether the women used it [34].

### 3.4 Results of Risk of Bias Assessment

**FIGURE 3:**
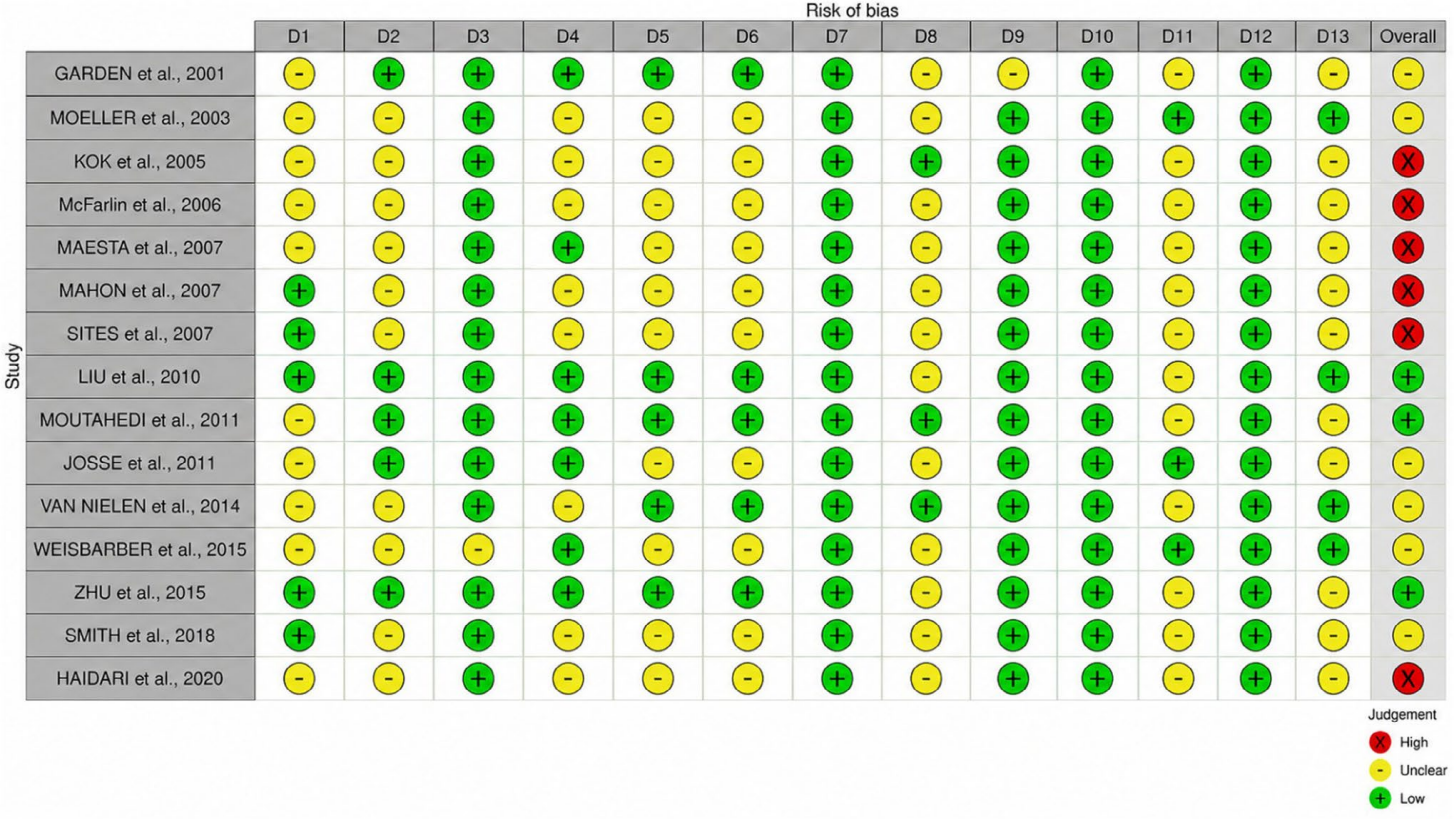
Risk of bias represented by each item assessed and the distribution of risk of bias across the studies included in the systematic review. D1: Was true randomization used for assignment of participants to treatment groups? D2: Was allocation to treatment groups concealed? D3: Were treatment groups similar at baseline? D4: Were participants blind to treatment assignment? D5: Were those delivering treatment blind to treatment assignment? D6: Were outcome assessors blind to treatment assignment? D7: Were treatment groups treated identically other than the intervention of interest? D8: Was follow‐up complete and, if not, were differences between groups in terms of follow‐up adequately described and analyzed? D9: Were participants analyzed in the groups to which they were randomized? D10: Were outcomes measured in the same way for treatment groups? D11: Were outcomes measured in a reliable way? D12: Was appropriate statistical analysis used? D13: Was the trial design appropriate, and were any deviations from the standard RCT design accounted for in the conduct and analysis of the trial?

**FIGURE 4:**
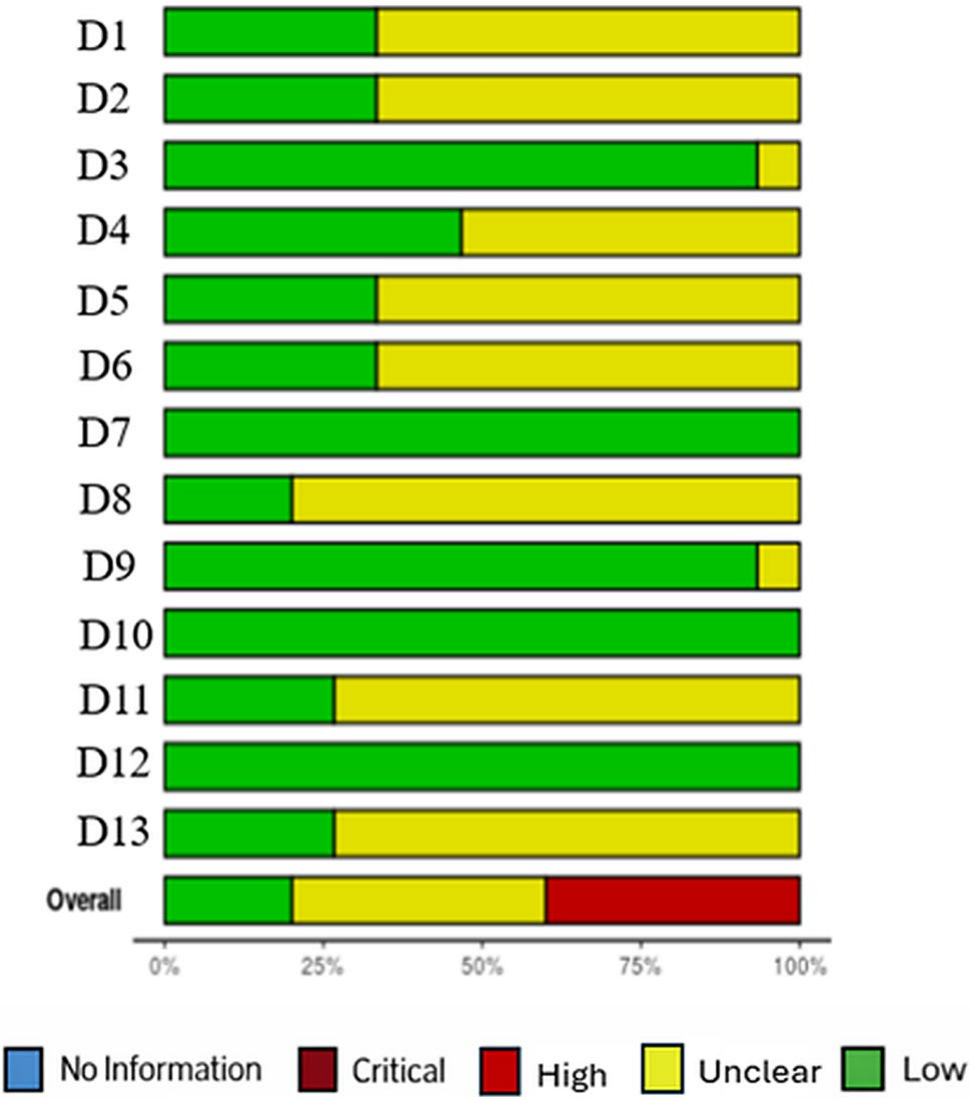
Distribution of risk of bias across the assessed domains for all included studies.

The methodological assessment of the 15 randomized clinical trials included, conducted using the JBI instrument involving 13 domains, covered aspects related to randomization, allocation, blinding, conduct, and analysis. Of the included studies, 40% provided insufficient information and presented a high risk of bias (*n* = 6), 40% presented an unclear/moderate risk of bias (*n* = 6), and 20% presented a low risk of bias (*n* = 3). The risk of bias for each study type is shown in Figures 3 and 4.

Overall, he noted that most studies presented uncertain risk, as indicated by yellow markings, and a high risk of bias, as indicated by red markings. Thus, methodological quality was considered moderate, with important limitations due to the absence of information or inadequate descriptions in several domains.

In domain D1 (randomization), most studies reported using some procedure for randomization; however, 10 studies did not describe the method used, resulting in an uncertain risk. Domain D2 (allocation concealment) also had a high number of studies with an uncertain rating, as there was no adequate detail on how the allocation was concealed, making it impossible to ensure that the process avoided selection bias. Comparability between groups at baseline (D3) was well reported in the studies, determining a low risk of bias in this domain. However, the domains related to blinding (D4 and D5) presented an uncertain risk due to the interventions (diets, supplements, and exercise programs), which makes complete blinding unfeasible. Domain D6 (blinding of outcome assessors) predominantly presented an uncertain risk since 10 studies did not report in detail how the assessors were aware of the allocation of groups. Domain D7, referring to identical treatment between groups except for the intervention, was met by all 15 studies, resulting in a low risk of bias.

In areas related to participant follow‐up, D8 was found to present an uncertain risk, as 12 studies did not provide sufficient detail on the procedure for following up on losses. On the other hand, domain D9 was assessed as low risk, since it adequately described sample losses and presented plausible justifications for these occurrences. Domains D10 and D11, related to consistent and reliable measurement of outcomes, also presented low risk, since the methods for measuring body composition were standardized and validated. Finally, domains D12 (adequacy of statistical analysis) and D13 (adequacy of design and compliance with RCT design) were found to be low risk, although some studies presented incomplete information. However, although some domains presented low risk, the predominance of uncertain classifications and the presence of high‐risk domains partially limit the methodology of the studies included in this review.

## 4 Discussion

**FIGURE 5:**
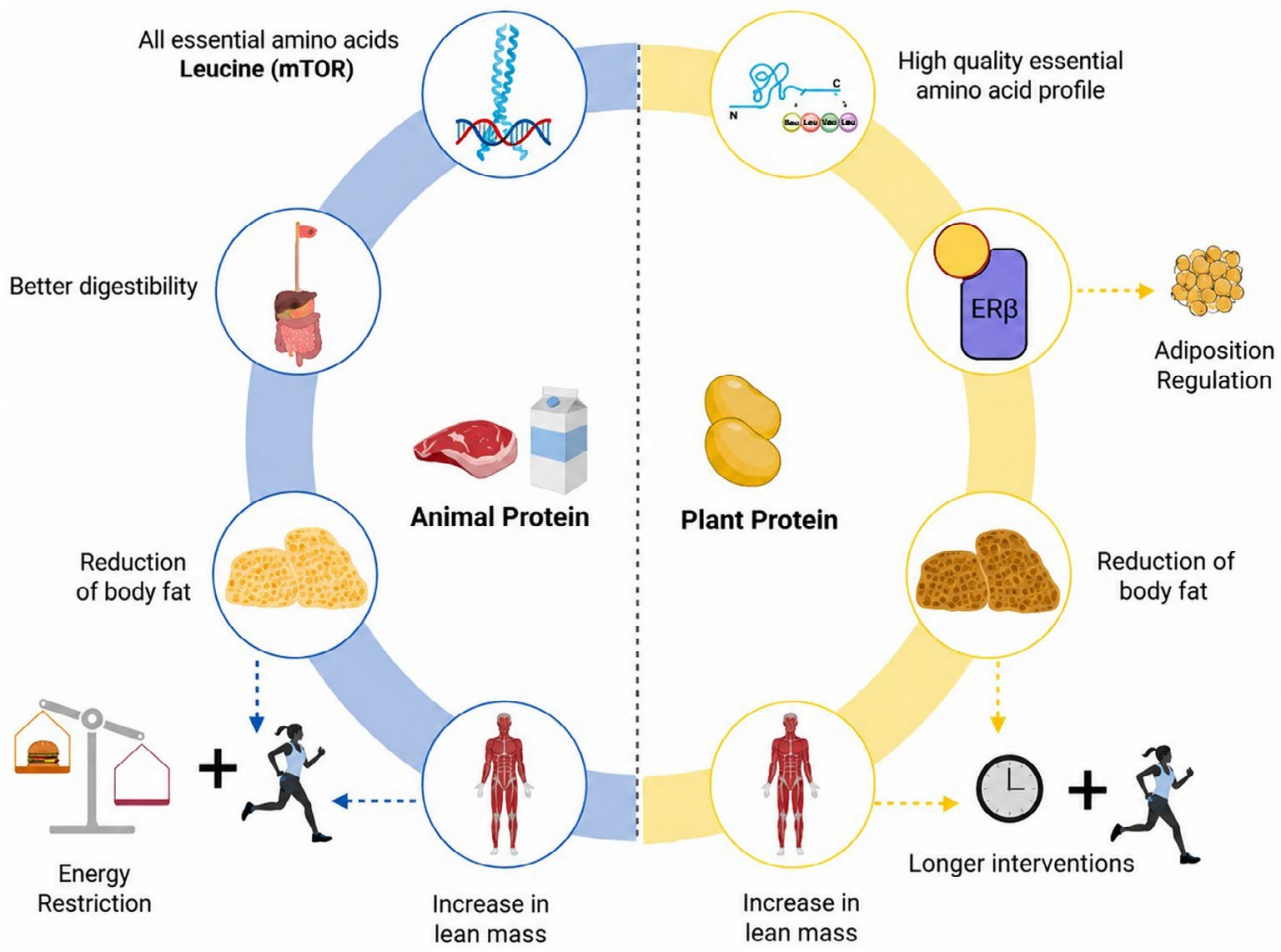
Summary of the review comparing animal and plant‐based proteins and their potential physiological effects. Animal‐based proteins (meat, dairy, and whey) generally have higher digestibility, bioavailability, and leucine content, and have shown more consistent results in promoting increased lean mass and reduced body fat, especially when combined with energy restriction and/or physical exercise. In contrast, plant‐based proteins (soy) tend to have more heterogeneous effects, although they can also contribute to the improvement of anthropometric markers and body composition, especially when combined with physical exercise. In general, the observed effects depend on the context of the intervention, including energy balance, physical exercise, and the dose and duration of protein intake, and the isolated effect of protein supplementation appears to be limited.

This review summarizes the effects of protein intake on anthropometric and body composition markers in pre‐ and postmenopausal women (Figure 5). In general, diets with higher protein intake from meat, milk, and whey showed more consistent improvements in these markers, especially when combined with weight‐loss strategies, even in the absence of structured physical exercise programs. In contrast, trials using soy protein showed greater heterogeneity in results, with more evident positive effects in long‐term interventions, with high doses, or when combined with other factors, such as physical exercise.

The results regarding animal protein are more consistent and may be directly associated with intrinsic differences between protein types. Whey protein, meat, and dairy products exhibit greater digestibility, bioavailability, and rapid absorption. Furthermore, essential amino acids are found in higher quantities, especially leucine, recognized as the main activator of the mTOR pathway, which is responsible for the activation and triggering of muscle protein synthesis [46, 47, 48, 49, 50]. However, the highlighted effects are more pronounced when aligned with a favorable metabolic context: energy restriction and physical exercise.

The findings regarding the increase in muscle mass resulting from the consumption of animal protein are more consistent and may be directly linked to intrinsic differences between protein types. Whey protein, meat, and dairy products exhibit higher digestibility, bioavailability, and rapid absorption. In addition, essential amino acids are found in greater quantities, especially leucine, recognized as the main activator of the mTOR pathway, which is responsible for activating and triggering muscle protein synthesis [46, 47, 48, 49, 50]. However, these highlighted effects are best pronounced when aligned with a favorable metabolic context, such as energy restriction and physical exercise.

Similarly, two studies included in this review [34, 36] evaluated dietary interventions that compared different meat sources with energy restriction. These interventions led to improvements in body weight and fat mass, but reduced lean body mass in the groups, regardless of the source of animal protein administered, reinforcing the finding that, under conditions of negative energy balance, protein quality appears to have a lesser influence, with energy balance being the primary determinant of changes in body composition. Furthermore, even in short‐term interventions, low‐calorie diets appear to be the primary driver of fat loss, while the preservation of lean body mass is not consistently observed across groups, despite increased protein intake. This effect is consistent with recent evidence suggesting that animal‐derived proteins may promote greater satiety and mitigate the loss of lean body mass during weight loss; however, these effects are limited under conditions of energy restriction, underscoring the predominant role of energy balance [51, 52, 53].

Supplementation with dairy protein (whey and milk protein) has been linked to improvements in body composition, particularly an increase in lean body mass. Studies involving the intake of 30 g/day of this protein [43, 45] reported an increase in lean body mass, a result also observed in other clinical trials [54, 55]. However, the findings should be interpreted with caution due to methodological variations and differences in physical activity control.

In the study by [45], although no structured exercise intervention was conducted, participants were assessed for their levels of physical activity (low to high intensity), which may have influenced the observed outcomes. Similarly, [56] demonstrated that whey protein supplementation can promote gains in lean body mass, even when used alone; however, these effects are enhanced when combined with resistance exercise protocols. In turn, [43], when evaluating protein supplementation in isolation over 2 years, observed an initial increase in lean body mass followed by stabilization, suggesting that the effect of protein may not be sustained over time in the absence of additional stimuli.

From a physiological point of view, whey protein is highly digestible and contains a high concentration of leucine, making it effective in stimulating muscle protein synthesis both at rest and after exercise. However, according to [46], the anabolic response depends not only on protein quality but also on the presence of an adequate mechanical stimulus, which may be particularly relevant for women in the post‐protein menopause, who are more sensitive to anabolism and have a higher risk of muscle loss. In fact, [40] reported an increase in lean mass in the group that consumed a higher amount of protein during an intervention involving milk protein combined with an exercise protocol with energy restriction (aerobic and resistance training). However, the combination of interventions does not allow the effect to be attributed solely to protein intake, reinforcing the need to consider multiple factors. Consistently, a recent meta‐analysis has shown that the effects on lean mass are more robust when protein supplementation is combined with physical training, highlighting the role of mechanical stimulation in muscle protein synthesis. This is because muscle contraction during exercise, especially in resistance training, promotes the activation of intracellular signaling pathways that converge to mTOR activation, increasing muscle protein synthesis [57, 58].

Regarding plant‐based protein, soy is widely used due to the presence of bioactive components such as isoflavones (genistein, daidzein, and glycitein), peptides, and globulins. Isoflavones, particularly daidzein and genistein, are among the most studied phytoestrogens because they are structurally similar to 17β‐estradiol, the main estrogen in humans [59, 60]. In vivo, they can interfere with hormone‐regulated pathways by competing with endogenous estrogens for receptor binding sites. In addition to interacting with estrogen receptors, phytoestrogens can also exert biological effects through mechanisms not mediated by estrogen receptors, including the modulation of cell signaling pathways, gene expression, and enzymatic activities [61]. In this review, we identified three studies that reported improvements in body composition and anthropometric markers, such as reduced BMI, weight, visceral fat, and abdominal fat, with the consumption of soy combined with isoflavones [35, 37, 38]. The isoflavone component can bind to two types of estrogen receptors present in adipose tissue: the α (ERα) and β (ERβ) receptors. Moreover, β is also present in skeletal muscle, in which isoflavones have a greater affinity for this subtype. Thus, isoflavones may regulate adiposity through estrogen receptor‐dependent mechanisms, corroborating the results of the studies reported [62]. Therefore, unlike whey protein, which acts directly on muscle mass synthesis, the effects of soy protein appear to occur through other mechanisms, primarily hormonal ones, and tend to depend more on being combined with physical exercise to promote gains in lean body mass.

On the other hand, the results included a study that showed that soy supplementation, when combined with physical exercise, can contribute to more efficient results, such as increased lean mass, similar to that observed with the consumption of animal protein, such as milk and whey protein supplementation [35]. A possible explanation for this finding is the high quality of soy‐based plant protein sources, whose essential amino acid (EAA) profile, particularly with respect to branched‐chain amino acids, including leucine, allows adequate stimulation of muscle protein synthesis when consumed in sufficient amounts and combined with physical exercise [63, 64]. A 2019 study compared the effects of whey and soy protein ingestion and demonstrated that both activate the mTORC1 signaling pathway, largely through leucine availability, such that rates of muscle protein synthesis do not differ between these protein sources following exercise [65]. Therefore, the improvements observed in the studies are due to the combined effect of consuming this protein source and exercising, and not merely to the isolated effect of soy protein on body composition.

Protein doses varied considerably, from amounts below current recommendations to amounts above those recommended for menopausal women. International guidelines suggest that women in this phase should consume between 1.0 and 1.2 g/kg/day to maintain lean mass, reaching 1.2 to 1.6 g/kg/day when losing weight, at risk of sarcopenia, or in the presence of this condition [12, 13, 14, 15]. Some studies involving whey or milk protein supplementation reached levels close to or above these recommended values, justifying the more consistent effects on body composition [38, 40, 41]. In contrast, interventions with soy protein used relatively lower fixed doses, so that, when adjusted for body weight, not all corresponded to the recommended intake [33, 35, 37, 38, 41]. This discrepancy may explain the heterogeneity of results observed in studies using vegetable protein and further reinforces the importance of considering body weight‐based recommendations when prescribing protein interventions, especially for this population.

Furthermore, the studies analyzed did not use hormone replacement therapy (HRT), as this could be a modifying factor in body composition. Evidence from some randomized controlled clinical trials indicates that HRT can attenuate increases in body fat, in addition to promoting small reductions in BMI and total fat mass [27, 30, 66, 67, 68]. Thus, its exclusion enables a more accurate evaluation of the effects of protein intake on body composition and anthropometric markers in these women.

This study has some important strengths. First, it included women at different stages of the climacteric, from pre‐ to post‐menopause, and with different nutritional statuses (normal weight, overweight, and obesity), resulting in a sample of 1161 participants and 15 randomized clinical trials. The most consistent effects on women with overweight or obesity, with reductions in weight and body fat, especially when protein supplementation, regardless of source, was associated with calorie restriction. Another relevant aspect is that most of the included studies used gold‐standard methods for body composition assessment, such as DXA, air‐displacement plethysmography, and MRI, which provide greater accuracy for outcomes related to lean mass, total body fat, and fat distribution.

However, some limitations should also be considered. The interventions vary widely in duration, type, and protein dose, which may have contributed to the heterogeneity of the results. Moreover, the inclusion of participants with varying baseline nutritional status may have influenced the observed responses, as changes in body composition following protein supplementation can vary with initial body weight. Individuals with overweight or obesity may also experience greater reductions in fat mass, especially when combined with energy restriction, while increases in lean mass may be less pronounced or more dependent on other stimuli, such as resistance training. Additionally, individuals with a healthy weight may experience smaller changes in body fat but may be more efficient at gaining lean body mass. Another relevant point is the predominance of studies conducted with postmenopausal women, with a scarcity of evidence in the pre‐ and postmenopausal phases, possibly due to greater hormonal variability in these phases, which hinders experimental control and the conduct of trials. Furthermore, the methodological quality of the studies was considered moderate, with 80% presenting a moderate or high risk of bias. This limitation stems mainly from the lack of information on randomization, blinding, and participant follow‐up. However, this scenario is expected in interventions involving diet and physical exercise, in which complete blinding is not feasible.

Despite these limitations, this review expands the current evidence that animal protein, when combined with physical exercise and/or energy restriction, tends to have more consistent effects on improving body composition and anthropometric markers in menopausal women than isolated consumption of vegetable protein. These findings highlight the importance of combining diet, exercise, and adequate protein supplementation as an effective strategy for managing body composition in women at this stage of life.

## 5 Conclusion

Different protein sources and increased protein intake may influence anthropometric and body composition markers in women during the climacteric period. However, these effects depend on various factors. Animal proteins showed more consistent results, possibly due to their composition and association with energy restriction and physical exercise, while plant proteins demonstrated more heterogeneous effects, with more evident benefits when combined with physical exercise. Thus, the isolated effect of protein supplementation tends to be limited.

In this sense, it is important to consider the woman's individuality, as well as the type of protein, dose, duration of intervention, presence of physical exercise, and energy restriction when establishing nutritional recommendations for body composition changes in women at different stages of life. Additionally, more research is needed to refine strategies targeting body composition in this population, especially studies that include women at other stages, since most available evidence focuses on postmenopausal women. Therefore, the results may contribute to the development of nutritional strategies to improve body composition and prevent associated changes during this period.

## Author Contributions

S.N.F. and M.L.d.S.F. designed and developed the research and contributed to the writing of the manuscript. A.C.P.K. assisted with the study methodology and search strategy planning. A.C.P.K., H.H.M.H., and S.A.V.R. contributed throughout the manuscript development process, with contributions to methodology, results presentation, and discussion development. All authors participated in the revision of the manuscript.

## Funding

This systematic review was funded by the Coordination for the Improvement of Higher Educational Personnel (CAPES) Foundation (Ministry of Education, Brazil, Financial code 001). H.H.M. Hermsdorff is a CNPq Research Productivity Fellow.

## Conflicts of Interest

The authors declare no conflicts of interest.

## Supporting information


**Table S1:** Detailed search strategy used in the systematic review.

## Data Availability

Data sharing is not applicable to this article as no datasets were generated or analyzed during the current study.
