# Supplementary material for: Dietary Intake of Animal and Plant‐Based Protein on Adiposity Measurements and Body Composition in Pre‐ and Postmenopausal Women: A Systematic Review of Randomized Clinical Trials
Source: FASEB J. 2026 Aug 1;40(15):e71977. doi: 10.1096/fj.202600792R (PMC13428612; doi:10.1096/fj.202600792R)
Supplement: Supplementary file 1 — Table S1: Detailed search strategy used in the systematic review. [file FSB2-40-e71977-s001.docx]

**SUPPLEMENTARY MATERIAL**

**Supplementary Table 1. Detailed search strategy used in the systematic review**

| **Database** |  | **Search strategies** | **Number of articles** |
| --- | --- | --- | --- |
| **PUBMED** | **#1** | "Menopause"[Mesh] OR "Premenopause"[Mesh] OR "Pre-menopausal Period" OR "Pre-Menopause" OR "Premenopausal Period" OR "Perimenopause"[Mesh] OR "Postmenopause"[Mesh] OR "Post-menopausal Period" OR "Period,Post-menopausal" OR "Post menopausal Period" OR "Post-Menopause" OR "Post Menopause" OR "Post-Menopauses" OR "Postmenopausal Period" OR "Period, Postmenopausal" OR "Climacteric"[Mesh] OR "Climacterics" OR "Change of Life" OR "Life Change" OR "Life Changes" | 96.402 results |
|  | **#2** | "Plant Proteins, Dietary"[Mesh] OR "Dietary Plant Protein" OR "Plant Protein, Dietary" OR "Dietary Plant Proteins" OR "Vegetable Proteins" OR "Proteins, Vegetable" OR "Protein, Vegetable" OR "Vegetable Protein" OR "Vegetable protein supplement" OR “Animal Proteins, Dietary"[Mesh] OR “Animal Protein, Dietary” OR “Dietary Animal Protein” OR “Dietary Animal Proteins” OR “Protein, Dietary Animal” OR “Proteins, Dietary Animal” | 111.374 results |
|  | **#3** | "Body Weight"[Mesh] OR “Body Weights” OR “Weight, Body” OR “Weights, Body” OR “hip circumference" OR "Waist Circumference"[Mesh] OR “Circumferences, Waist” OR “Circumference, Waist” OR “Waist Circumferences” OR “neck circumference” OR "Waist-Hip Ratio"[Mesh] OR “Ratios, Waist-Hip” OR “Ratio, Waist-Hip” OR “Waist Hip Ratio” OR “Waist-Hip Ratios” OR “Waist-to-Hip Ratio” OR “Ratios, Waist-to-Hip” OR “Ratio, Waist-to-Hip” OR “Waist to Hip Ratio” OR “Waist-to-Hip Ratios” OR “Body Mass” OR "Body Fat Distribution"[Mesh] OR “Distribution, Body Fat” OR “Fat Distribution, Body” OR “Body Fat Patterning” OR “Fat Patterning, Body” OR “Patterning, Body Fat” OR "Adipose Tissue"[Mesh] OR “Tissue, Adipose” OR “Fatty Tissue” OR “Tissue, Fatty” OR “Fat Pad” OR “Fat Pads” OR “Pad, Fat” OR “Pads, Fat” OR “Body Fat” OR “Fat mass” OR “body fat percentage” | [9](https://pubmed.ncbi.nlm.nih.gov/?term=%22Body+Weight%22%5BMesh%5D+OR+%E2%80%9CBody+Weights%E2%80%9D+OR+%E2%80%9CWeight%2C+Body%E2%80%9D+OR+%E2%80%9CWeights%2C+Body%E2%80%9D+OR+%E2%80%9Chip+circumference%22+OR+%22Waist+Circumference%22%5BMesh%5D+OR+%E2%80%9CCircumferences%2C+Waist%E2%80%9D+OR+%E2%80%9CCircumference%2C+Waist%E2%80%9D+OR+%E2%80%9CWaist+Circumferences%E2%80%9D+OR+%E2%80%9Cneck+circumference%E2%80%9D+OR+%22Waist-Hip+Ratio%22%5BMesh%5D+OR+%E2%80%9CRatios%2C+Waist-Hip%E2%80%9D+OR+%E2%80%9CRatio%2C+Waist-Hip%E2%80%9D+OR+%E2%80%9CWaist+Hip+Ratio%E2%80%9D+OR+%E2%80%9CWaist-Hip+Ratios%E2%80%9D+OR+%E2%80%9CWaist-to-Hip+Ratio%E2%80%9D+OR+%E2%80%9CRatios%2C+Waist-to-Hip%E2%80%9D+OR+%E2%80%9CRatio%2C+Waist-to-Hip%E2%80%9D+OR+%E2%80%9CWaist+to+Hip+Ratio%E2%80%9D+OR+%E2%80%9CWaist-to-Hip+Ratios%E2%80%9D+OR+%E2%80%9CBody+Mass%E2%80%9D+OR+%22Body+Fat+Distribution%22%5BMesh%5D+OR+%E2%80%9CDistribution%2C+Body+Fat%E2%80%9D+OR+%E2%80%9CFat+Distribution%2C+Body%E2%80%9D+OR+%E2%80%9CBody+Fat+Patterning%E2%80%9D+OR+%E2%80%9CFat+Patterning%2C+Body%E2%80%9D+OR+%E2%80%9CPatterning%2C+Body+Fat%E2%80%9D+OR+%22Adipose+Tissue%22%5BMesh%5D+OR+%E2%80%9CTissue%2C+Adipose%E2%80%9D+OR+%E2%80%9CFatty+Tissue%E2%80%9D+OR+%E2%80%9CTissue%2C+Fatty%E2%80%9D+OR+%E2%80%9CFat+Pad%E2%80%9D+OR+%E2%80%9CFat+Pads%E2%80%9D+OR+%E2%80%9CPad%2C+Fat%E2%80%9D+OR+%E2%80%9CPads%2C+Fat%E2%80%9D+OR+%E2%80%9CBody+Fat%E2%80%9D&sort=&size=200)41.784 results |
|  |  | **#1 AND #2 AND #3**  ("Menopause"[Mesh] OR "Premenopause"[Mesh] OR "Pre-menopausal Period" OR "Pre-Menopause" OR "Premenopausal Period" OR "Perimenopause"[Mesh] OR "Postmenopause"[Mesh] OR "Post-menopausal Period" OR "Period,Post-menopausal" OR "Post menopausal Period" OR "Post-Menopause" OR "Post Menopause" OR "Post-Menopauses" OR "Postmenopausal Period" OR "Period, Postmenopausal" OR "Climacteric"[Mesh] OR "Climacterics" OR "Change of Life" OR "Life Change" OR "Life Changes") AND ("Plant Proteins, Dietary"[Mesh] OR "Dietary Plant Protein" OR "Plant Protein, Dietary" OR "Dietary Plant Proteins" OR "Vegetable Proteins" OR "Proteins, Vegetable" OR "Protein, Vegetable" OR "Vegetable Protein" OR "Vegetable protein supplement" OR “Animal Proteins, Dietary"[Mesh] OR “Animal Protein, Dietary” OR “Dietary Animal Protein” OR “Dietary Animal Proteins” OR “Protein, Dietary Animal” OR “Proteins, Dietary Animal”) AND ("Body Weight"[Mesh] OR “Body Weights” OR “Weight, Body” OR “Weights, Body” OR “hip circumference" OR "Waist Circumference"[Mesh] OR “Circumferences, Waist” OR “Circumference, Waist” OR “Waist Circumferences” OR “neck circumference” OR "Waist-Hip Ratio"[Mesh] OR “Ratios, Waist-Hip” OR “Ratio, Waist-Hip” OR “Waist Hip Ratio” OR “Waist-Hip Ratios” OR “Waist-to-Hip Ratio” OR “Ratios, Waist-to-Hip” OR “Ratio, Waist-to-Hip” OR “Waist to Hip Ratio” OR “Waist-to-Hip Ratios” OR “Body Mass” OR "Body Fat Distribution"[Mesh] OR “Distribution, Body Fat” OR “Fat Distribution, Body” OR “Body Fat Patterning” OR “Fat Patterning, Body” OR “Patterning, Body Fat” OR "Adipose Tissue"[Mesh] OR “Tissue, Adipose” OR “Fatty Tissue” OR “Tissue, Fatty” OR “Fat Pad” OR “Fat Pads” OR “Pad, Fat” OR “Pads, Fat” OR “Body Fat” OR “Fat mass” OR “body fat percentage”) | 80 results |
|  | | | |
| **EMBASE** | **#1** | 'menopause'/exp OR 'menopausal age' OR 'menopausal female' OR 'menopausal woman' OR 'menopause' OR 'menopause age' OR 'menopauze' OR 'climacterium'/exp OR 'climacteric' OR 'climacterium' OR 'menopausal transition' OR 'perimenopausal female' OR 'perimenopausal woman' OR 'perimenopause' OR 'postmenopause'/exp OR 'post menopause' OR 'postmenopausal female' OR 'postmenopausal period' OR 'postmenopausal women' OR 'postmenopause' | 188.922 results |
|  | **#2** | 'protein intake'/exp OR 'animal proteins, dietary' OR 'diet protein' OR 'dietary animal proteins' OR 'dietary egg proteins' OR 'dietary fish proteins' OR 'dietary plant proteins' OR 'dietary protein' OR 'dietary proteins' OR 'egg proteins, dietary' OR 'fish proteins, dietary' OR 'food protein' OR 'intake, protein' OR 'plant proteins, dietary' OR 'protein consumption' OR 'protein feeding' OR 'protein food' OR 'protein intake' OR 'protein nutrition' OR 'plant protein'/exp OR 'fruit protein' OR 'fruit proteins' OR 'grain protein' OR 'grain proteins' OR 'leaf protein' OR 'nut protein' OR 'nut proteins' OR 'plant protein' OR 'plant proteins' OR 'protein, vegetable' OR 'vegetable protein' OR 'vegetable proteins' | 256.779 results |
|  | **#3** | 'body weight'/exp OR 'body weight' OR 'total body weight' OR 'weight, body' OR 'hip circumference'/exp OR 'hip circumference' OR 'waist circumference'/exp OR 'waist circumference' OR 'waist size' OR 'neck circumference'/exp OR 'neck circumference' OR 'waist hip ratio'/exp OR 'hip to waist ratio' OR 'hip waist ratio' OR 'waist hip ratio' OR 'waist to hip ratio' OR 'waist-hip ratio' OR 'body mass'/exp OR 'bmi (body mass index)' OR 'quetelet index' OR 'body ban mass' OR 'body mass' OR 'body mass index' OR 'body fat percentage'/exp OR 'body fat distribution'/exp OR 'adipose tissue distribution' OR 'body fat distribution' OR 'fat tissue distribution' OR 'fatty tissue distribution' OR 'subcutaneous fat distribution' OR 'visceral fat distribution' OR 'fat mass'/exp OR 'fat mass' | 1.887.389 results |
|  |  | **#1 AND #2 AND #3** | 495 results |
|  | | | |
| **COCHRANE LIBRARY** | **#1** | Menopause OR Premenopause OR Pre-menopausal Period OR Pre-Menopause OR Premenopausal Period OR Perimenopause OR Postmenopause OR Post-menopausal Period OR Period,Post-menopausal OR Post menopausal Period OR Post-Menopause OR Post Menopause OR Post-Menopauses OR Postmenopausal Period OR Period, Postmenopausal OR Climacteric OR Climacterics OR Change of Life OR Life Change OR Life Changes | 104.820 results |
|  | **#2** | Plant Proteins, Dietary OR Dietary Plant Protein OR Plant Protein, Dietary OR Dietary Plant Proteins OR Vegetable Proteins OR Proteins, Vegetable OR Protein, Vegetable OR Vegetable Protein OR Vegetable protein supplement OR Animal Proteins, Dietary OR Animal Protein, Dietary OR Dietary Animal Protein OR Dietary Animal Proteins OR Protein, Dietary Animal OR Proteins, Dietary Animal | 3.117 results |
|  | **#3** | Body Weight OR Body Weights OR Weight, Body OR Weights, Body OR hip circumference OR Waist Circumference OR Circumferences, Waist OR Circumference, Waist OR Waist Circumferences OR neck circumference OR Waist-Hip Ratio OR Ratios, Waist-Hip OR Ratio, Waist-Hip OR Waist Hip Ratio OR Waist-Hip Ratios OR Waist-to-Hip Ratio OR Ratios, Waist-to-Hip OR Ratio, Waist-to-Hip OR Waist to Hip Ratio OR Waist-to-Hip Ratios OR Body Mass OR Body Fat Distribution OR Distribution, Body Fat OR Fat Distribution, Body OR Body Fat Patterning OR Fat Patterning, Body OR Patterning, Body Fat OR Adipose Tissue OR Tissue, Adipose OR Fatty Tissue OR Tissue, Fatty OR Fat Pad OR Fat Pads OR Pad, Fat OR Pads, Fat OR Body Fat OR Fat mass OR body fat percentage | 164.245 results |
|  |  | **#1 AND #2 AND #3**  (Menopause OR Premenopause OR Pre-menopausal Period OR Pre-Menopause OR Premenopausal Period OR Perimenopause OR Postmenopause OR Post-menopausal Period OR Period,Post-menopausal OR Post menopausal Period OR Post-Menopause OR Post Menopause OR Post-Menopauses OR Postmenopausal Period OR Period, Postmenopausal OR Climacteric OR Climacterics OR Change of Life OR Life Change OR Life Changes) AND (Plant Proteins, Dietary OR Dietary Plant Protein OR Plant Protein, Dietary OR Dietary Plant Proteins OR Vegetable Proteins OR Proteins, Vegetable OR Protein, Vegetable OR Vegetable Protein OR Vegetable protein supplement OR Animal Proteins, Dietary OR Animal Protein, Dietary OR Dietary Animal Protein OR Dietary Animal Proteins OR Protein, Dietary Animal OR Proteins, Dietary Animal) AND (Body Weight OR Body Weights OR Weight, Body OR Weights, Body OR hip circumference OR Waist Circumference OR Circumferences, Waist OR Circumference, Waist OR Waist Circumferences OR neck circumference OR Waist-Hip Ratio OR Ratios, Waist-Hip OR Ratio, Waist-Hip OR Waist Hip Ratio OR Waist-Hip Ratios OR Waist-to-Hip Ratio OR Ratios, Waist-to-Hip OR Ratio, Waist-to-Hip OR Waist to Hip Ratio OR Waist-to-Hip Ratios OR Fat mass OR body fat percentage) AND (Placebos OR normal protein diet OR Protein-Restricted OR Diet, Protein Restricted OR Diet, Low-Protein OR Diet, Low Protein OR Diets, Low-Protein OR Low-Protein Diets OR Low-Protein Diet OR Low Protein Diet OR Protein-Restricted Diet OR Diets, Protein-Restricted OR Protein Restricted Diet OR Protein-Restricted Diets OR Diet, Protein-Free OR Diet, Protein Free OR Diets, Protein-Free OR Protein-Free Diet OR Protein-Free Diets OR control group) | 217 results |
